# Mitochondrial dysfunction and oxidative stress in patients with chronic kidney disease

**DOI:** 10.14814/phy2.12780

**Published:** 2016-05-09

**Authors:** Jorge L. Gamboa, Frederic T. Billings, Matthew T. Bojanowski, Laura A. Gilliam, Chang Yu, Baback Roshanravan, L. Jackson Roberts, Jonathan Himmelfarb, T. Alp Ikizler, Nancy J. Brown

**Affiliations:** ^1^ Division of Clinical PharmacologyVanderbilt University Medical CenterNashvilleTennessee; ^2^ Department of AnesthesiologyVanderbilt University Medical CenterNashvilleTennessee; ^3^ Department of PhysiologyEast Carolina UniversityGreenvilleNorth Carolina; ^4^ Department of BiostatisticsVanderbilt University Medical CenterNashvilleTennessee; ^5^ Kidney Research InstituteUniversity of WashingtonSeattleWashington; ^6^ Division of NephrologyDepartment of MedicineVanderbilt University Medical CenterNashvilleTennessee

**Keywords:** BNIP3, chronic kidney disease, mitochondria, mitochondrial DNA copy number, oxidative stress, PGC1*α*, skeletal muscle

## Abstract

Mitochondria abnormalities in skeletal muscle may contribute to frailty and sarcopenia, commonly present in patients with chronic kidney disease (CKD). Dysfunctional mitochondria are also a major source of oxidative stress and may contribute to cardiovascular disease in CKD. We tested the hypothesis that mitochondrial structure and function worsens with the severity of CKD. Mitochondrial volume density, mitochondrial DNA (mtDNA) copy number, BNIP3, and PGC1*α* protein expression were evaluated in skeletal muscle biopsies obtained from 27 subjects (17 controls and 10 with CKD stage 5 on hemodialysis). We also measured mtDNA copy number in peripheral blood mononuclear cells (PBMCs), plasma isofurans, and plasma F2‐isoprostanes in 208 subjects divided into three groups: non‐CKD (eGFR>60 mL/min), CKD stage 3–4 (eGFR 60–15 mL/min), and CKD stage 5 (on hemodialysis). Muscle biopsies from patients with CKD stage 5 revealed lower mitochondrial volume density, lower mtDNA copy number, and higher BNIP3 content than controls. mtDNA copy number in PBMCs was decreased with increasing severity of CKD: non‐CKD (6.48, 95% CI 4.49–8.46), CKD stage 3–4 (3.30, 95% CI 0.85–5.75, *P* = 0.048 vs. non‐CKD), and CKD stage 5 (1.93, 95% CI 0.27–3.59, *P* = 0.001 vs. non‐CKD). Isofurans were higher in patients with CKD stage 5 (median 59.21 pg/mL, IQR 41.76–95.36) compared to patients with non‐CKD (median 49.95 pg/mL, IQR 27.88–83.46, *P* = 0.001), whereas F2‐isoprostanes did not differ among groups. Severity of CKD is associated with mitochondrial dysfunction and markers of oxidative stress. Mitochondrial abnormalities, which are common in skeletal muscle from patients with CKD stage 5, may explain the muscle dysfunction associated with frailty and sarcopenia in CKD. Further studies are required to evaluate mitochondrial function in vivo in patients with different CKD stages.

## Introduction

Frailty and sarcopenia are commonly present in patients with CKD and they are associated with increased morbidity and mortality in this population (Bao et al. 2012; Isoyama et al. 2014). Frailty is a phenotype that requires the presence of at least three of the following characteristics: unintentional weight loss, self‐reported exhaustion or fatigue, measured muscle weakness, slow walking speed, or low physical activity (Morley et al. 2013). Approximately 73% percent of patients are frail at the time of initiation of dialysis (Bao et al. 2012). Sarcopenia, defined as reduction in muscle mass or reduced muscle strength/physical performance (Cooper et al. 2012), is one of the components of the frailty phenotype and it is also associated with increased mortality in patients with end‐stage renal disease (Isoyama et al. 2014).

Cardiovascular morbidity and mortality is also higher in patients with CKD compared to the general population (Foley et al. 1998; Sarnak and Levey 1999). The probability of cardiovascular events increases dramatically when the estimated glomerular filtration rate (eGFR) is lower than 45 mL/min/1.73 m^2^ (Go et al. 2004). Traditional risk factors such as high cholesterol or smoking do not explain the increased cardiovascular morbidity in CKD. Rather, increased oxidative stress and systemic inflammation, which are present in CKD, have been proposed as additional risk factors for cardiovascular events in patients with CKD (Himmelfarb et al. 2002).

Mitochondria abnormalities in skeletal muscle may explain the frailty and sarcopenia observed in CKD. Previous studies have shown that patients with CKD exhibit skeletal muscle mitochondrial dysfunction compared to healthy individuals (Durozard et al. 1993; Thompson et al. 1993; Conjard et al. 1995; Pastoris et al. 1997; Kemp et al. 2004). Briefly, these studies showed decreased activity of mitochondrial enzymes, such as citrate synthase and hydroxyacyl‐CoA dehydrogenase, and the prolongation of phosphocreatine recovery time after exercise in skeletal muscle in patients with CKD. A recent study in mice also suggested that reduction in skeletal muscle mitochondria number would precede the onset of sarcopenia (Tamaki et al. 2014); however, no study has measured mitochondrial number in human skeletal muscle from patients with CKD.

Mitochondrial dysfunction is associated with increased oxidative stress and inflammation (Ide et al. 2001; López‐Armada et al. 2013). Several studies have shown an association between mitochondrial dysfunction and atherosclerosis (Ballinger et al. 2002; Madamanchi and Runge 2007), suggesting that impaired mitochondrial function may also contribute to the pathogenesis of cardiovascular diseases. While earlier studies showed mitochondrial abnormalities in peripheral blood mononuclear cells (PBMCs) from patients with CKD (Granata et al. 2009; Rao et al. 2009), no study has extensively examined the association among the severity of CKD, mitochondrial function, and oxidative stress.

Thus, we examined vastus lateralis muscle biopsies from patients with CKD stage 5 undergoing maintenance hemodialysis (MHD) for the presence of ultrastructural and mitochondrial abnormalities using electron microscopy. We tested the hypothesis that mitochondrial volume density (i.e., mitochondrial number) decreases in skeletal muscle from patients with CKD. We also tested the hypothesis that mitochondrial function decreases with the severity of CKD. To this end, we measured mitochondrial DNA (mtDNA) copy number from PBMCs, lactate concentrations, and the ratio of plasma isofurans to F2‐isoprostanes in patients at different stages of CKD. We used isofurans as biomarker of oxidative stress associated with mitochondrial function because previous studies have shown that isofurans formation is favored over F2‐isoprostanes formation when oxygen tissue tension is high, as observed in the setting of mitochondrial dysfunction (Fessel et al. 2002). Using an animal model of mitochondrial lipid peroxidation we also verified that the induction of mitochondrial dysfunction increases the production of isofurans relative to F2‐isoprostanes.

## Subjects and Methods

### Subjects

#### Skeletal muscle biopsy group

Skeletal muscle biopsies for analysis of mitochondrial ultrastructure by electron microscopy were obtained from 11 patients with CKD stage 5 on MHD and 17 controls. Patients with CKD were clinically stable and had been dialyzed three times per week for four hours at the time of enrollment. Control subjects had normal kidney function at the time of enrollment and had similar self‐reported activity and age compared to the patients with CKD.

#### Severity of CKD group

Blood samples for isolation of PBMCs and measurement of isofurans and F2‐isoprostanes were obtained from participants in a clinical trial of patients on MHD (NCT00878969) and a trial of subjects undergoing cardiac surgery (NCT00791648). All measurements on the subjects scheduled for elective cardiac surgery were performed prior to surgery. These subjects were stable and not experiencing acute changes in renal function or metabolic homeostasis. Blood was obtained from 208 patients: 109 subjects with eGFR > 60 mL/min/1.73 m^2^ (non‐CKD), 36 subjects with eGFR between 60 and 15 mL/min/1.73 m^2^ (CKD 3–4), and 63 subjects with eGFR < 15 mL/min/1.73 m^2^ on MHD (CKD 5/MHD). Glomerular filtration rate was estimated using the Modification of Diet in Renal Disease (MDRD) formula. All the subjects on hemodialysis were clinically stable and had been dialyzed three times per week for four hours and were on MHD for at least 6 months.

All the studies were approved by the Vanderbilt University Institutional Review Board for Research on Human Subjects, and all subjects provided written informed consent.

### Muscle biopsies

Biopsies were obtained from the vastus lateralis skeletal muscle. The biopsies from controls with normal kidney function or from patients with CKD stage 5 on MHD were obtained under aseptic conditions and local lidocaine anesthesia in the Clinical Research Center (CRC) at Vanderbilt or at the University of Kentucky CRC by percutaneous needle biopsy. After removing subcutaneous fat, biopsies were immersed in fixatives for electron microscopy. Muscle biopsies from patients with CKD stage 5 were obtained on a nondialysis day.

### Transmission electron microscopy

Muscle biopsies were dissected and cut into small pieces and fixed in glutaraldehyde (4%) and paraformaldehyde (2%) for 2 h at room temperature. Samples were prepared for electron microscopy as previously described (Gamboa and Andrade 2012). Briefly, samples were postfixed in osmium tetraoxide (1%) for 1 h, dehydrated and embedded for further sectioning. Thin (80 nm) fiber transverse sections were stained with uranyl acetate and lead citrate and examined with a transmission electron microscope.

### Mitochondrial volume density

Approximately 40 thin sections per subject were used for the calculation of mitochondrial volume density in skeletal muscle using the stereological point counting method as previously described (Broskey 2013). A 20 × 20 grid (corresponding to 0.25 *μ*m^2^) was superimposed on electron‐micrographs captured at 11,000 magnification. Mitochondrial volume was calculated as the percentage of points of the grid that intersect any mitochondria.

### Western blot analysis

Skeletal muscle samples were homogenized in the presence of proteases inhibitors and heated at 95°C with denaturing Laemli buffer. Samples (30 *μ*g) were resolved electrophoretically in 4–20% acrylamide gel and transferred to polyvinylidene difluoride membranes (Immobilon‐FL, Millipore, Billerica, MA). After blocking the membranes with Odyssey^®^ blocking buffer, we performed overnight incubation with rabbit polyclonal antibodies against BCL‐2/adenovirus interacting protein 3 (BNIP3, Cell Signaling, Danvers, MA) and peroxisome proliferator activated‐receptor gamma coactivator 1 alpha (PGC1*α*, abcam, Cambridge, MA). Membranes were then incubated for 1 hour with Alexa Fluor 680‐conjugated goat anti‐rabbit IgG (Invitrogen, Carlsbad, CA). Membranes were scanned with Odyssey^®^ Infrared Imaging System (LI‐COR Biosciences, Lincoln, NE). Band densities were analyzed using NIH Image J software. Coomassie blue staining was used as loading control.

### Peripheral blood mononuclear cells (PBMCs) isolation

PBMCs were isolated using Ficoll‐Paque^™^ gradient. For this purpose, whole blood (2 mL) was mixed with Hank's buffered salt solution (HBSS; 1/1 ratio – volume/volume) and placed on top of Ficoll‐Paque^™^ (3 mL) in 15 mL conical tubes. After centrifuging for 45 min at 500 g, the layer containing PBMCs (between the plasma and Ficoll‐Paque^™^) was removed. Cells were then washed with HBSS and centrifuged at 200 g for 10 min. Finally, PBMCs were resuspended in the appropriate media and frozen at −80°C until further processing.

### Mitochondrial DNA copy number

DNA was extracted using Gentra^®^ Puragene^®^ extraction kit according to the manufacturers. Mitochondrial copy number was calculated by measuring the amount mitochondrial DNA relative to the amount of nuclear DNA. To do so, the mitochondrial encoded NADH dehydrogenase 1 (ND1) gene, the nuclear lipoprotein lipase (LPL) for PBMCs, and the nuclear B2M gene for skeletal muscle were amplified using real‐time PCR (SYBR green). Fluorescence signal was detected using a Bio‐Rad iCycler. The PCR reactions were performed in triplicate for each gene. Standard curves of 3, 30, 300, 3,000, and 30,000 nuclear genome equivalents for each gene were included in each run.

### Lactate determination

Plasma lactate levels were measured using a colorimetric/fluorimetric kit (Biovision, Milpitas, CA) in samples obtained from arterial blood only.

### Isoprostanes and isofurans

Both were measured in plasma separated from EDTA‐anticoagulated blood using negative ion gas‐chromatography mass spectroscopy (GC/MS) as previously described (Morrow et al. 1990; Fessel et al. 2002; Milne et al. 2007).

### Animal model of mitochondrial dysfunction

Kidneys from mice treated with doxorubicin were obtained from investigators at Eastern Carolina University following Institutional Animal Care and Use Committee approval. C57BL/6NJ ovariectomized female mice received a single IP injection of doxorubicin (20 mg/kg) or vehicle. Seventy‐two hours later, kidneys were collected from animals, snap frozen in liquid nitrogen, and stored at −80°C until further processing. Kidney F2‐isoprostanes and isofurans were measured by GC/MS as previously described (Morrow et al. 1990; Fessel et al. 2002; Milne et al. 2007). We also measured complex I activity was measured in kidney mitochondria. For this purpose, mitochondria were isolated by differential centrifugation. Briefly, kidneys were homogenized in a buffer containing 20 mmol/L Tris/MOPS, 400 mmol/L sucrose, and 2 mmol/L EGTA. After initial centrifugation at 750 g, supernatants were collected and centrifuged at 8,000 g for 10 min. Pellet was then resuspended and centrifuged again at 8,000 g for 10 min. Crude mitochondria pellet was then resuspended, protein concentration measured using BCA method and adjusted to 5 mg/mL of protein. Complex I activity was detected using a microplate assay kit (ab109721, abcam^®^, Cambridge, MA) according to the manufacturer instructions, which measures the oxidation of NADH to NAD^+^.

### Statistical analysis

Standard graphing and screening techniques were used to detect any outliers and to ensure data accuracy. The distributions of continuous endpoints were examined for normality using Kolmogorov–Smirnov test. In the case of non‐normally distributed data, logarithmic data transformations were performed or nonparametric tests were used. General lineal model was used to compare the different groups and contrasts for specific two‐group comparisons were implemented as appropriate. Age and race were included as covariates for the analysis of mitochondrial volume density in skeletal muscle. Gender, body mass index (BMI), age and diagnosis of hypertension, and diagnosis of diabetes were included as covariates in the rest of the analyses. Additional two‐group comparisons were also performed using two‐sample T‐tests or Wilcoxon rank sum tests. Hypotheses were tested at the level of *α* = 0.05. SPSS^®^ v22 (IBM, North Carolina) was used for the analyses.

## Results

### Ultrastructural abnormalities in skeletal muscle mitochondria in patients with CKD stage 5 on MHD

**Figure 1:**
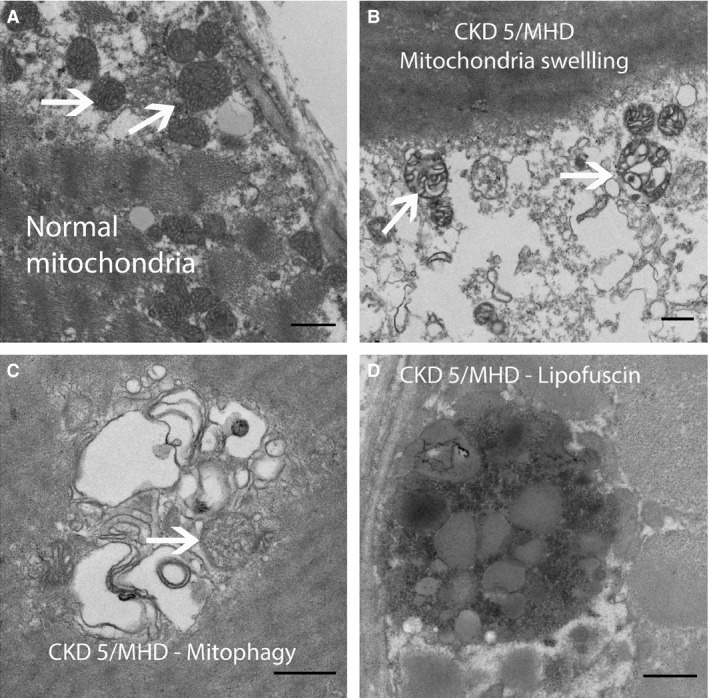
Representative electron micrograph of skeletal muscles from a healthy control (A) and patients with CKD stage 5 on maintenance hemodialysis (CKD 5/MHD). (A) Normal subsarcolemmal mitochondria. (B) Mitochondria with signs of swelling. (C) Double membrane structure compatible with auto‐phagosome. (D) Lipofuscin pigment (scale bar = 500 *μ*m).

Table 1 shows the demographic characteristic of controls and patients with CKD stage 5 in the skeletal muscle biopsy group. Age, body mass index (BMI), gender, mean arterial blood pressure (MAP), and heart rate were similar between the groups. There was a greater proportion of African American in patients with CKD stage 5 than in controls. We examined the structural changes in skeletal muscle mitochondria between controls and patients with CKD stage 5 on MHD. Compared to controls (Fig. 1A), in patients undergoing MHD we found mitochondria demonstrating signs of cristae swelling (Fig. 1B), and double membrane structures compatible with auto‐phagosomes, some of them surrounding mitochondria (Fig. 1C). These findings suggest that mitophagy, a particular type of autophagy unique to mitochondria, is increased in patients with CKD stage 5 on MHD. Intracellular lipofuscin granules (Fig. 1D) were also abundant in patients undergoing MHD, indicating oxidative damage in mitochondria and lysosomes.

**Table 1:** Demographic and clinical characteristics: skeletal muscle biopsy group

Parameter	Control (*n* = 17)	CKD5/MHD (*n* = 10)	*P* value
Age (years)	52.9 ± 8.1	50.5 ± 14.3	0.63
Gender (male)	7/17 (41.2%)	7/10 (70%)	0.15
Race (African American)	3/17 (17.6%)	8/10 (80%)	0.002
Body mass index (kg/m^2^)	30.4 ± 6.6	29.1 ± 4.7	0.56
History of diabetes	0/17 (0%)	2/10 (20%)	0.13
MAP (mmHg)	87.2.9 ± 12.4	101.5 ± 23.4	0.12
Heart rate (bpm)	65.7 ± 8.2	73.8 ± 13.2	0.13

Data are presented as mean ± SD. MAP, mean arterial blood pressure.

### Mitochondrial volume density and mitochondrial DNA copy number in skeletal muscle are decreased in patients with CKD stage 5 on MHD

**Figure 2:**
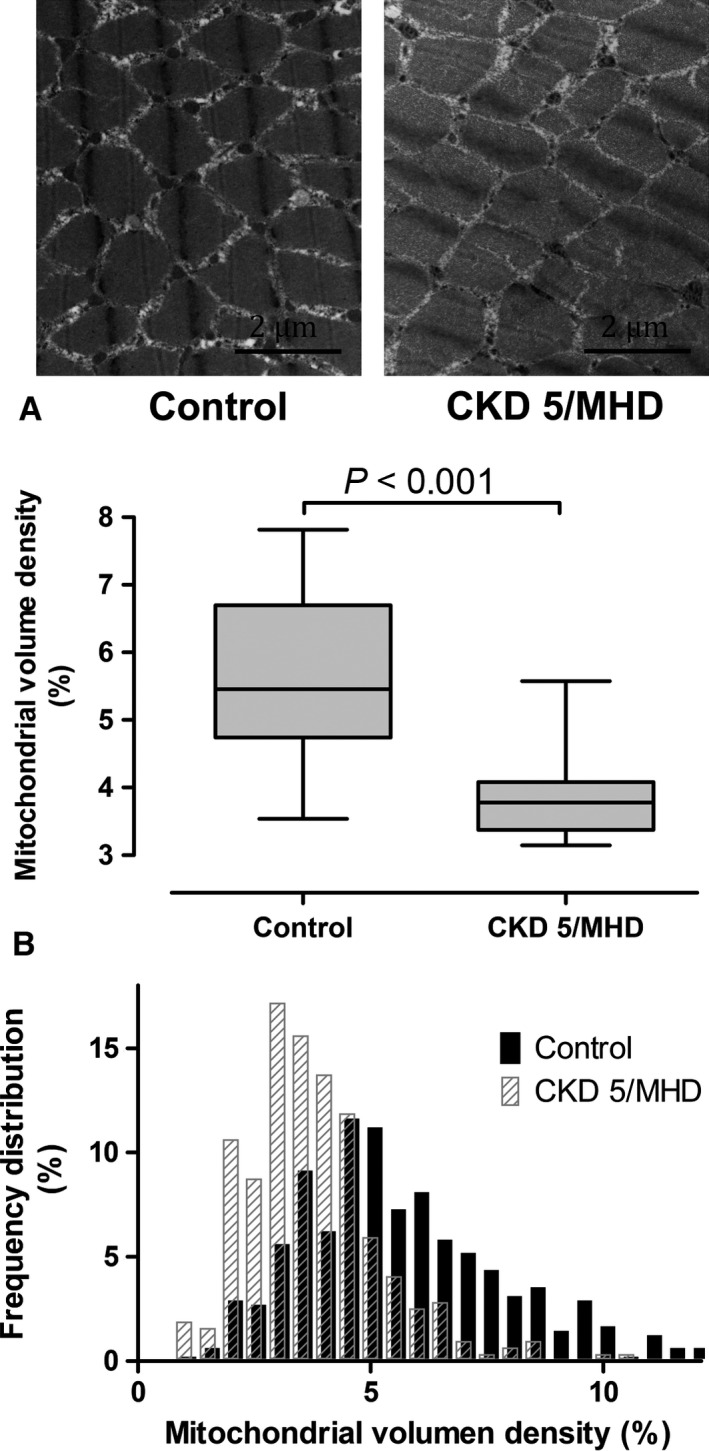
(A) Mitochondrial volume density (MVD) in controls with normal kidney function (*N* = 17) and patients with CKD stage 5 on maintenance hemodialysis (CKD 5/MHD,* N* = 10) in the vastus lateralis. (B) Frequency distribution of MVD in individual myofibers showed a greater proportion of fibers with lower MVD in patients with CKD stage 5.

**Figure 3:**
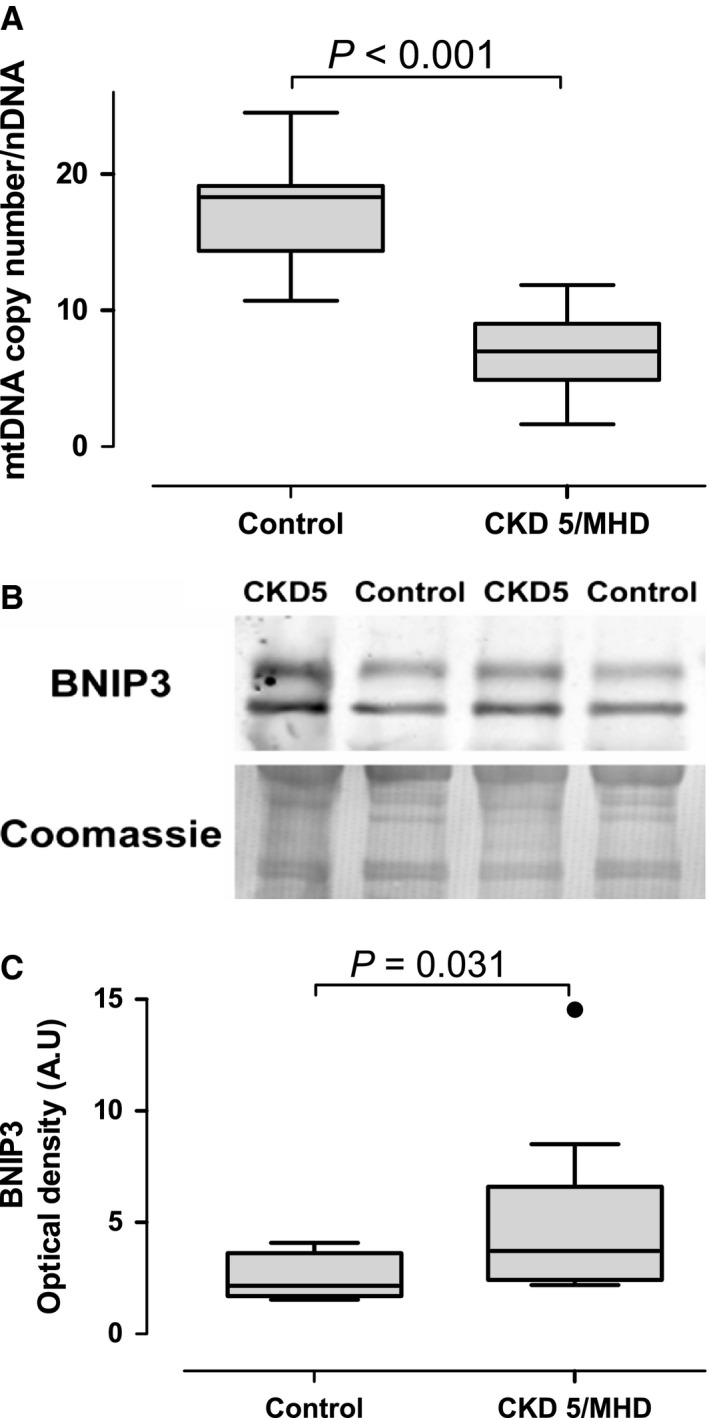
(A) Mitochondrial DNA (mtDNA) copy number in the vastus lateralis in controls with normal kidney function (*N* = 8) and patients with CKD stage 5 on maintenance hemodialysis (CKD5/MHD,* N* = 10). (B) Representative Western blots of BCL‐2/adenovirus interacting protein 3 BNIP3 in the vastus lateralis in controls and patients with CKD5/MHD (*N* = 9 in each group); Coomassie blue staining was used as loading control. (C) Optical densities were calculated using the NIH software image J.

Mitochondrial volume density, evaluated with stereological methods, was significantly lower in patients with CKD stage 5 compared to controls. There was no effect of race on mitochondrial volume density and the difference in mitochondrial volume density between patients with CKD and controls remained highly significant after adjusting for race and age (Fig. 2, *P* = 0.002). Frequency distribution of mitochondrial volume density in individual myofibers showed a shift to the left in patients undergoing MHD, indicating a greater proportion of fibers with lower mitochondrial volume density (Fig. 2). We also found that mitochondrial volume density decreased as the age increased in the controls (*ρ *= −0.65, *P* = 0.005). There was no relationship between age and mitochondrial volume density in patients with CKD stage 5 on MHD. We also found decreased mitochondrial DNA copy number in skeletal muscles from patients with CKD stage 5 on MHD (Fig. 3A). Western blot analysis showed that BNIP3, an inducer of mitophagy, was elevated in patients skeletal muscles from patients with CKD stage 5 compared to controls (Fig. 3B). There was no difference in PGC1*α* protein expression in skeletal muscles from patients with CKD stage 5 compared to controls (*P* = 0.387).

### Mitochondrial DNA copy number in PBMCs and the stage of CKD

Table 2 shows the demographic characteristic of the three groups based on the severity of CKD. Of note, subjects with CKD stage 5 on MHD were younger and had a higher prevalence of diabetes. Therefore, all the analyses were adjusted for age and history of diabetes. The percentage of African American subjects was also higher in CKD patients on MHD compared to the small proportion in the other two groups. Because there were only one or two African American subjects in these groups we could not adjust for race. Within subjects with CKD stage 5 on MHD, race did not affect any of the study outcomes, however.

**Table 2:** Demographic and clinical characteristics: CKD severity group

Parameter	Non‐CKD (*n* = 109)	CKD 3–4 (*n* = 36)	CKD 5/MHD (*n* = 63)	*P* value
Age (years)	54.6 ± 12.2	60.9 ± 10.9	53.9 ± 12.0	0.01
Gender (male)	76/109 (69.7%)	19/36 (52.8%)	38/63 (59.4%)	0.08
Race (African American)	2/109 (1.8%)	1/36 (2.8%)	41/63 (64.1%)	<0.001
Smoker	31/109 (28.4%)	7/36 (19.4%)	20/63 (31.7%)	0.4
Body mass index (kg/m^2^)	29.9 ± 7.7	30.3 ± 5.8	30.6 ± 8.2	0.8
History of hypertension	87/109 (80.6%)	33/36 (91.7%)	55/63 (87.3%)	0.2
History of diabetes	29/109 (26.6%)	15/36 (41.7%)	33//63 (52.4%)	0.001
Systolic blood pressure (mmHg)	125.3 ± 18.9	125.6 ± 17.7	149.2 ± 25.3 (pre) 127.4 ± 18.6 (post)	<0.001 0.8

Data are presented as mean ± SD. Pre indicates predialysis and post indicates postdialysis.

**Figure 4:**
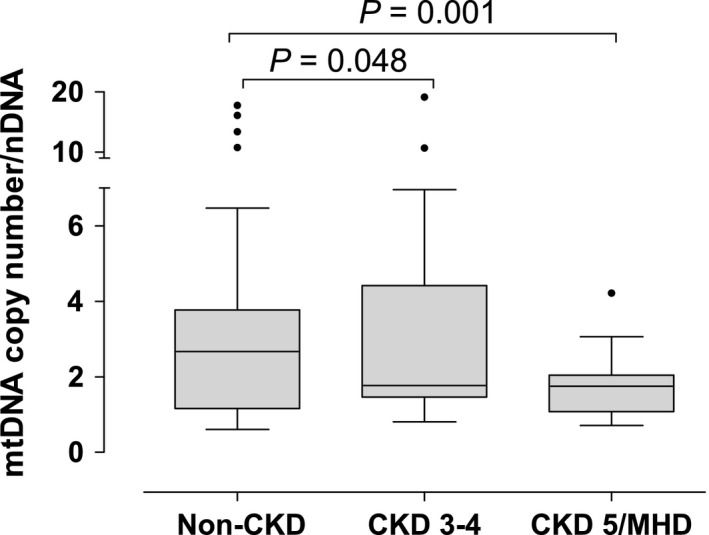
Mitochondrial DNA (mtDNA) copy number in peripheral blood mononuclear cells (PBMCs) in patients with different stages of CKD. Patients were divided into three groups according to eGFR: Non‐CKD (eGFR > 60 mL/min, *N* = 28), CKD 3–4 (eGFR between 15 and 60 mL/min, *N* = 17), and CKD 5/MHD (eGFR < 15 mL/min on maintenance hemodialysis, *N* = 42). *P* values were adjusted for gender, age, BMI, and history of diabetes.

We isolated PBMCs from patients at different stages of CKD in order to evaluate the association between severity of CKD and mitochondrial DNA copy number. Mitochondrial DNA copy number was progressively lower with increased severity of kidney disease after adjusting by age, gender, and BMI, as well as presence or absence of hypertension and history of diabetes (Fig. 4).

### Plasma lactate levels and the stage of CKD

**Figure 5:**
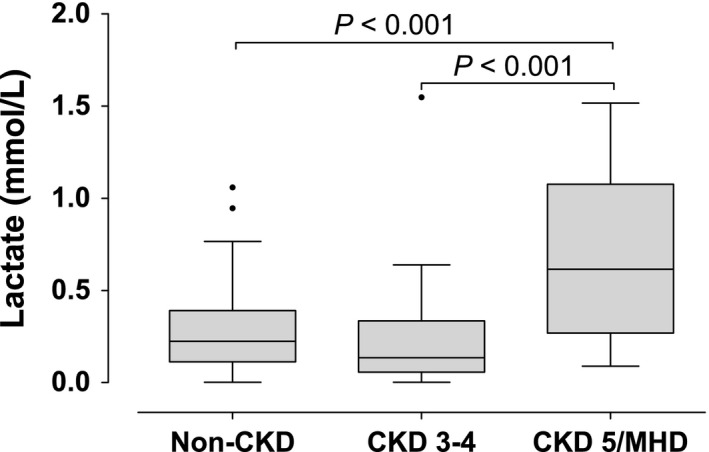
Plasma lactate levels in patients with different stages of chronic kidney disease (CKD). Patients were divided in three groups according to eGFR: Non‐CKD (eGFR > 60 mL/min, *N* = 109), CKD 3–4 (eGFR between 15 and 60 mL/min, *N* = 35), and CKD 5/MHD (eGFR < 15 mL/min on maintenance hemodialysis, *N* = 42). *P* values were adjusted for gender, age, BMI, and history of diabetes. Normal lactate concentrations are less than 2 mmol/L.

Sensitive but not specific to mitochondrial dysfunction, elevations in plasma lactate are widely used as a marker of anaerobic metabolism induced by mitochondrial dysfunction. We evaluated plasma lactate levels in the three groups of subjects. There was no difference in lactate levels between subjects with non‐CKD and CKD stages 3–4 (Fig. 5). Patients with CKD stage 5 on MHD had higher lactate levels compared to the other two groups (Fig. 5, *P* < 0.001). There was a trend toward inverse correlation between lactate and mitochondrial DNA copy number that was not statistical significance (*P* = 0.087)

### Isofurans to F2‐isoprostanes ratio as a biomarker of mitochondrial dysfunction in vivo

**Figure 6:**
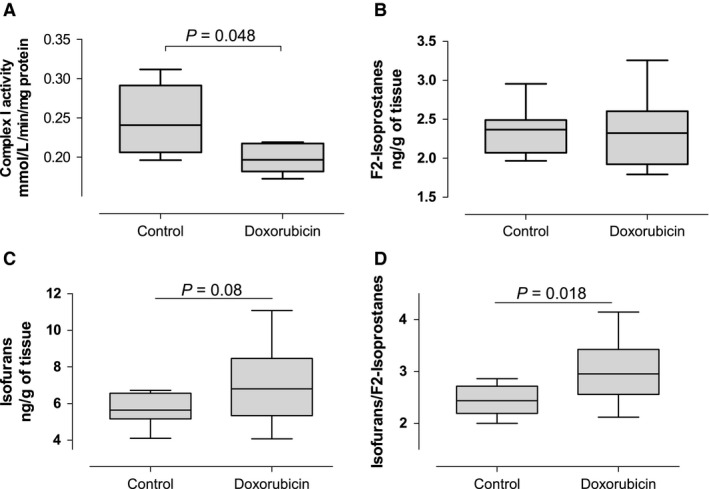
Mitochondrial complex I activity, F2‐isoprostanes, and isofurans in kidneys from mice treated with doxorubicin. Mice were treated with a single IP injection of either vehicle (*N* = 10) or doxorubicin (20 mg/kg, *N* = 10). Kidneys were collected 72 h later. Complex I activity was normalized to the amount of protein in mitochondria pellets.

F2‐isoprostanes and isofurans are both products of nonenzymatic oxidation of arachidonic acid and have emerged as highly sensitive and specific measures of oxidative stress in vivo (Kadiiska et al. 2005). Under conditions of high oxygen tissue tension isofurans are preferentially formed. This may occur due to increased oxygen delivery (breathing a high FIO2) or due to decreased oxygen consumption (as in mitochondrial dysfunction). Thus, the ratio of isofurans to F2‐isoprostanes have been proposed as markers of mitochondrial dysfunction (Fessel et al. 2003). To assess the validity of isofurans formation as a marker of mitochondrial dysfunction in vivo, we measured isofurans and F2‐isoprostanes in kidneys of mice treated with doxorubicin, an anthracycline antibiotic and antineoplastic drug that induces mitochondrial lipid peroxidation resulting in mitochondrial dysfunction and oxidative damage. In isolated mitochondria, Complex I (NADH:ubiquinone oxidoreductase) activity was lower in kidneys from mice treated with doxorubicin (Fig. 6A, *P* = 0.048), confirming the presence of doxorubicin‐induced mitochondrial dysfunction. We found that mice treated with doxorubicin had similar levels of F2‐isoprostanes compared with mice treated with vehicle (Fig. 6B). Isofurans levels tended to be higher in mice treated with doxorubicin (Fig. 6C, *P* = 0.08), and the ratio of isofurans to F2‐isoprostanes was significantly higher in mice treated with doxorubicin (Fig. 6D, *P* = 0.018).

### Isofurans and F2‐isoprostanes plasma levels and the stage of CKD in humans

**Figure 7:**
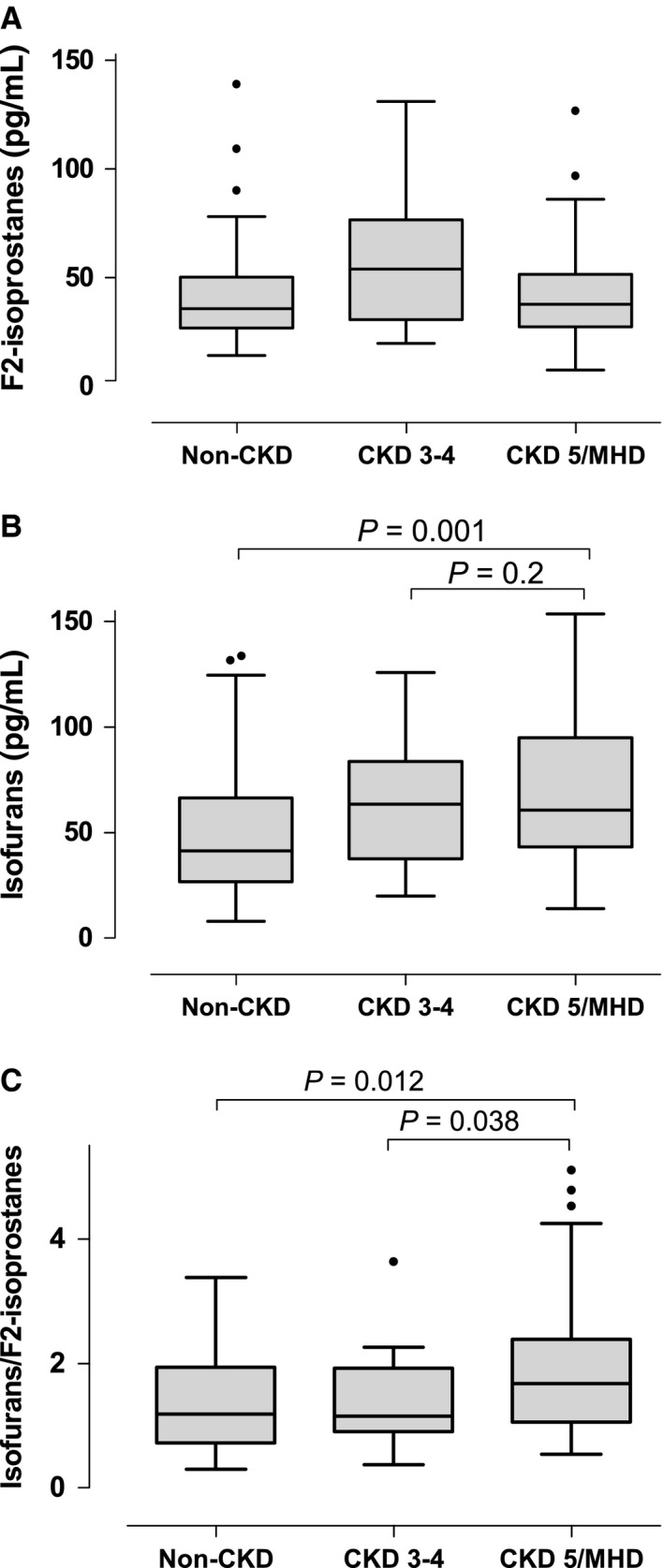
F2‐isoprostanes and isofurans in patients with different stages of chronic kidney disease (CKD). Patients were divided in three groups according to eGFR: Non‐CKD (eGFR > 60 mL/min, *N* = 74), CKD 3–4 (eGFR between 15 and 60 mL/min, *N* = 26), and CKD5/MHD (eGFR < 15 mL/min on maintenance hemodialysis, *N* = 61). *P* values were adjusted for gender, age, BMI, and history of diabetes. Normal values in healthy controls for F2‐isoprostanes and isofurans are 35 ± 6 pg/mL and 43 ± 5 pg/mL, respectively.

F2‐isoprostanes levels were similar among the groups (Fig. 7A). In contrast, concentrations of isofurans were higher in patients with CKD stage 5 on MHD compared with subjects with non‐CKD (*P* = 0.001), but not with subjects with CKD stages 3–4 (*P* = 0.2, Fig. 7B). Furthermore, the ratio of isofurans to F2‐isoprostanes was increased in patients with CKD stage 5 suggesting a preferential production of isofurans in patients undergoing MHD with severe CKD compared to patients with less severe CKD (Fig. 7C). We further evaluated the correlation between mitochondrial DNA copy number and markers of oxidative stress. There was no correlation among these variables in patients with non‐CKD or with CKD stages 3–4. In contrast, the ratio of isofurans to F2‐isprostanes increased as mtDNA decreased in patients with CKD stage 5 undergoing MHD (*ρ *= −0.38, *P* = 0.01).

## Discussion

This study tested the hypothesis that mitochondrial volume density in skeletal muscle decreases in patients with CKD stage 5 on MHD. We found that mitochondrial volume density and mitochondrial DNA copy number were decreased; whereas BNIP3, a marker of mitophagy, was increased in skeletal muscle in patients with CKD stage 5. We also found significant structural abnormalities in skeletal muscle mitochondria compared to individuals with normal kidney function. This study also tested the hypothesis that circulating biomarkers of mitochondrial dysfunction and oxidative stress increase with the severity of chronic kidney disease. We found that mitochondrial dysfunction, measured by mtDNA copy number, lactate, or isofurans concentration, is present and worse in patients with CKD stage 5 undergoing MHD compared to patients with non‐CKD and with earlier stages of CKD (i.e., stages 3 and 4). In addition, mtDNA copy number is decreased even in patients with CKD stage 3–4, suggesting that changes in mitochondrial DNA occur before the establishment of end‐stage renal disease. Finally, our results also indicate that plasma isofurans concentration and the ratio of isofurans to F2‐isoprostanes are potential biomarkers of mitochondrial dysfunction in vivo.

This is the first study to demonstrate decreased mitochondrial volume density in skeletal muscle in patients undergoing MHD. We also found that mitochondrial DNA copy number is decreased in the muscles of patients undergoing MHD. These observations are consistent with previous studies that showed reduced mitochondrial oxidative capacity and reduced activity of mitochondrial enzymes in skeletal muscle from patients with end‐stage renal disease (Adey et al. 2000; Lewis et al. 2012). A possible explanation for these findings is the increased mitophagy as indicated by increased BNIP3 content in skeletal muscles from patients on MHD. BNIP3 is a BH3‐only protein that is translocated to the outer mitochondrial membrane and induces mitophagy (Zhang et al. 2008), the cellular process that removes damaged mitochondria. Previous studies also showed changes in mitochondria morphology, myofibrillar degeneration, autophagic vacuoles, lipofuscin granules, and intracellular accumulation of glycogen and lipid (Ahonen 1980; Shah et al. 1983; Diesel et al. 1993; Lewis et al. 2012). We confirmed the presence of these abnormalities, particularly swollen mitochondria and autophagosomes, and lipofuscin pigments. The latter has been linked to decreased lysosomal function and is commonly present with aging (Terman et al. 2006, 2009; O'Leary et al. 2013). Thus, the skeletal muscle ultrastructure shows signs of accelerated aging in patient with CKD stage 5. Overall, the mitochondrial structural abnormalities could be one of the reasons leading to diminished physical performance and endurance observed in patients with CKD. It may also explain the increased prevalence of frailty and sarcopenia in this population. The etiology of these abnormalities is not clear, but a study in an animal model of CKD suggests that reduction in muscle mitochondria precedes the loss of muscle volume and muscle power (Tamaki et al. 2014). We found that skeletal muscle mitochondrial number is decreased in patients with CKD stage 5; however, further studies should evaluate if muscle mitochondrial reduction precedes the onset of sarcopenia in patients with CKD.

A previous study reported that mtDNA copy number in PBMCs is lower in patients on MHD than in healthy controls in individuals aged 50 years or older, and that mtDNA copy number in PBMCs predicts survival in patients with end‐stage renal disease undergoing MHD (Rao et al. 2009). We also found that mitochondrial DNA copy number in PBMCs is decreased in patients on MHD compared to controls with no CKD. Our study further suggests that mtDNA copy number in PBMCs is already decreased in patients with CKD stages 3–4. Mitochondrial DNA is more susceptible to damage by oxidative stress than nuclear DNA because of a lack of protection from histone proteins (Croteau and Bohr 1997). Mutations in mtDNA and reduced mtDNA copy number are commonly present in cells exposed to excessive concentration of reactive oxygen species (Fliss et al. 2000). Thus, mtDNA copy number has been proposed as a surrogate of mitochondrial function (Malik and Czajka 2013).

Lactate concentration reflects anaerobic metabolism, and is a crude measurement of mitochondrial dysfunction from low mitochondrial oxidative capacity. Resting lactate levels are high in obese individuals and correlate positively with blood pressure and insulin resistance (DiGirolamo et al. 1992; Baron et al. 1993; Crawford et al. 2008). A recent community‐based cohort study found that increased lactate levels were associated with higher risk of heart failure and all‐cause mortality (Matsushita et al. 2013). Interestingly, we found that lactate levels are higher in patients with CKD stage 5 on MHD, a population with high mortality rates and an increased incidence of heart failure. A previous study found that, in patients undergoing MHD, lactate levels correlate inversely with mitochondrial DNA copy number in PBMCs, suggesting the association between mitochondrial dysfunction and lactate levels (Rao et al. 2009). We also noted a trend toward an inverse association between lactate and mitochondrial DNA copy number but did not reach statistical significance. Nevertheless, more studies are required to study the relationship between mitochondrial dysfunction and lactate in patients on MHD.

The relative formation of either F2‐isoprostanes or isofurans depends on tissue oxygen tension. In conditions of high oxygen tension the formation of isofurans is favored (Fessel et al. 2002). This may occur due to decreased oxygen consumption (as occurs in mitochondrial dysfunction). We found that the levels of isofurans were higher in patients with CKD stage 5 on MHD than in patients with CKD stages 1–4. Furthermore, the increased ratio of isofurans to F2‐isoprostanes suggests a preferential production of isofurans in patients undergoing MHD. These findings may be a consequence of mitochondrial dysfunction, as previously hypothesized (Fessel et al. 2003). We tested this hypothesis in mice injected with doxorubicin, which is known to induce mitochondrial dysfunction (Berthiaume and Wallace 2007). Doxorubicin is an antineoplastic agent that interacts with the mitochondrial NADH dehydrogenase (complex I) forming a semiquinone radical (Berthiaume and Wallace 2007). Doxorubicin inhibits mitochondrial respiration and complex I activity (Yen et al. 1999; Gilliam et al. 2013). In accordance, we found that complex I activity is decreased in kidney mitochondria from mice treated with doxorubicin. We also found that kidney isofurans to F2‐isoprostanes ratios are increased in mice treated with doxorubicin, showing that doxorubicin‐induced mitochondrial dysfunction is associated with increased isofurans formation. Furthermore, we also found that the ratio of isofurans to F2‐isoprostanes correlates inversely with mitochondrial DNA copy number in patient on CKD stage 5. This finding suggests that the increased ratio reflect oxidative stress due to mitochondrial dysfunction in patients on MHD. Moreover, a recent dose‐escalation study found that supplementation with coenzyme Q_10_, which may improve mitochondrial function, decreased isofurans levels and the ratio of isofurans to F2‐isoprostanes in patients on MHD (Yeung et al. 2015). Further studies are required, however, to establish the specific sources of the overall oxidative stress burden in patients on MHD.

This study has some limitations. We did not evaluate mitochondrial function in skeletal muscle; however, due to the organization of mitochondrial cristae, mitochondrial ultrastructure correlates with mitochondrial function (Zick et al. 2009). Further studies should confirm the association between mitochondrial ultrastructure and function in skeletal muscle in patients with CKD. The study groups were not matched by race. Nevertheless, race did not affect any of the study outcomes in patients with CKD stage 5 on MHD. Furthermore, a previous study did not find association between race and mitochondrial DNA copy number in a cohort of patients with CKD stage 5 (Rao et al. 2009). Predialysis blood pressure was also significantly higher in patients with CKD stage 5 compared to non‐CKD and patients with CKD stage 3–4. Although we cannot exclude a confounding effect of hypertension, in a subgroup of patients that were matched by age, gender, race, and BMI, we found increased mtDNA copy number in hypertensive subjects without CKD compared to patients with CKD stage 5 (data not shown). Furthermore, we found no correlation between blood pressure and mtDNA copy number in each of the studied groups. These results suggest that elevated blood pressure does not affect mtDNA copy number. In contrast, the inclusion of controls with a high prevalence of cardiovascular disease, comparable to patients with CKD stage 5, may be considered as strength to avoid overestimation of the differences among the groups.

In summary, we found that patients with CKD stage 5 have decreased mitochondrial volume density in skeletal muscle than non‐CKD controls that may be explained by increased mitophagy. We also observed numerous ultrastructural abnormalities in muscles biopsies from patients with CKD stage 5 on MHD such as swollen mitochondria, liposfuscin pigments, and autophagosomes. We found that mitochondrial oxidative stress and dysfunction, assessed by in vivo biomarkers (i.e., mitochondrial DNA copy number in PBMCs, plasma lactate, and ratio of isofurans to F2‐isoprostanes), is increased in patients with CKD stage 5 on MHD compared to patients on CKD stage 3–4. Mitochondrial DNA copy number may be a more sensitive marker of mitochondrial function since it is already decreased by CKD stage 3–4. Our data in an animal model indicate that doxorubicin‐induced mitochondrial dysfunction is associated with increase in renal isofurans concentrations and an increase in the ratio of isofurans to F2‐isoprostanes suggesting that measurement of this ratio can be used to detect mitochondrial dysfunction in vivo. Additional studies are needed to validate the potential utility of novel in vivo biomarkers of mitochondrial dysfunction in CKD and interventions targeted at these abnormalities.

## Conflict of Interest

No conflicts of interest. The results from this manuscript have not been previously published, except in abstract format.

## References

[phy212780-bib-0001] Adey, D. , R. Kumar , J. T. McCarthy , and K. S. Nair . 2000 Reduced synthesis of muscle proteins in chronic renal failure. Am. J. Physiol. Endocrinol. Metab. 278:E219–E225. 10662705 10.1152/ajpendo.2000.278.2.E219

[phy212780-bib-0002] Ahonen, R. E. 1980 Striated muscle ultrastructure in uremic patients and in renal transplant recipients. Acta Neuropathol. 50:163–166. 6994424 10.1007/BF00692869

[phy212780-bib-0003] Ballinger, S. W. , C. Patterson , C. A. Knight‐Lozano , D. L. Burow , C. A. Conklin , Z. Hu , et al. 2002 Mitochondrial integrity and function in Atherogenesis. Circulation 106:544–549. 12147534 10.1161/01.cir.0000023921.93743.89

[phy212780-bib-0004] Bao, Y. , L. Dalrymple , G. M. Chertow , G. A. Kaysen , and K. L. Johansen . 2012 Frailty, dialysis initiation, and mortality in end‐stage renal disease. Arch. Intern. Med. 172:1071–1077. 22733312 10.1001/archinternmed.2012.3020 PMC4117243

[phy212780-bib-0005] Baron, A. D. , G. Brechtel‐Hook , A. Johnson , and D. Hardin . 1993 Skeletal muscle blood flow. A possible link between insulin resistance and blood pressure. Hypertension 21:129–135. 8428775 10.1161/01.hyp.21.2.129

[phy212780-bib-0006] Berthiaume, J. M. , and K. B. Wallace . 2007 Adriamycin‐induced oxidative mitochondrial cardiotoxicity. Cell Biol. Toxicol. 23:15–25. 17009097 10.1007/s10565-006-0140-y

[phy212780-bib-0007] Broskey, N. T. D. 2013 Skeletal muscle mitochondrial and lipid droplet content assessed with standardized grid sizes for stereology. J. Appl. Physiol. 115:765–770. 23788579 10.1152/japplphysiol.00063.2013

[phy212780-bib-0008] Conjard, A. , B. Ferrier , M. Martin , A. Caillette , H. Carrier , and G. Baverel . 1995 Effects of chronic renal failure on enzymes of energy metabolism in individual human muscle fibers. J. Am. Soc. Nephrol. 6:68–74. 7579072 10.1681/ASN.V6168

[phy212780-bib-0009] Cooper, C. , W. Dere , W. Evans , J. A. Kanis , R. Rizzoli , A. A. Sayer , et al. 2012 Frailty and sarcopenia: definitions and outcome parameters. Osteoporos. Int. 23:1839–1848. 22290243 10.1007/s00198-012-1913-1

[phy212780-bib-0010] Crawford, S. O. , M. S. Ambrose , R. C. Hoogeveen , F. L. Brancati , C. M. Ballantyne , and J. H. Young . 2008 Association of lactate with blood pressure before and after rapid weight loss. Am. J. Hypertens. 21:1337–1342. 18802433 10.1038/ajh.2008.282

[phy212780-bib-0011] Croteau, D. L. , and V. A. Bohr . 1997 Repair of oxidative damage to nuclear and mitochondrial DNA in mammalian cells. J. Biol. Chem. 272:25409–25412. 9325246 10.1074/jbc.272.41.25409

[phy212780-bib-0012] Diesel, W. , B. K. Knight , T. D. Noakes , C. R. Swanepoel , van Zyl Smit R. , R. O. C. Kaschula , et al. 1993 Morphologic features of the myopathy associated with chronic renal failure. Am. J. Kidney Dis. 22:677–684. 8238013 10.1016/s0272-6386(12)80430-6

[phy212780-bib-0013] DiGirolamo, M. , F. D. Newby , and J. Lovejoy . 1992 Lactate production in adipose tissue: a regulated function with extra‐adipose implications. FASEB J. 6:2405–2412. 1563593 10.1096/fasebj.6.7.1563593

[phy212780-bib-0014] Durozard, D. , P. Pimmel , S. Baretto , A. Caillette , M. Labeeuw , G. Baverel , et al. 1993 31P NMR spectroscopy investigation of muscle metabolism in hemodialysis patients. Kidney Int. 43:885–892. 8479125 10.1038/ki.1993.124

[phy212780-bib-0015] Fessel, J. P. , N. A. Porter , K. P. Moore , J. R. Sheller , and L. J. Roberts . 2002 Discovery of lipid peroxidation products formed in vivo with a substituted tetrahydrofuran ring (isofurans) that are favored by increased oxygen tension. Proc. Natl Acad. Sci. U. S. A. 99:16713–16718. 12482927 10.1073/pnas.252649099 PMC139209

[phy212780-bib-0016] Fessel, J. P. , C. Hulette , S. Powell , L. J. Roberts , and J. Zhang . 2003 Isofurans, but not F2‐isoprostanes, are increased in the substantia nigra of patients with Parkinson's disease and with dementia with Lewy body disease. J. Neurochem. 85:645–650. 12694390 10.1046/j.1471-4159.2003.01709.x

[phy212780-bib-0017] Fliss, M. S. , H. Usadel , O. L. Caballero , L. Wu , M. R. Buta , S. M. Eleff , et al. 2000 Facile detection of mitochondrial DNA mutations in tumors and bodily fluids. Science 287:2017–2019. 10720328 10.1126/science.287.5460.2017

[phy212780-bib-0018] Foley, R. N. , P. S. Parfrey , and M. J. Sarnak . 1998 Epidemiology of cardiovascular disease in chronic renal disease. J. Am. Soc. Nephrol. 9:S16–S23. 11443763

[phy212780-bib-0019] Gamboa, J. L. , and F. H. Andrade . 2012 Muscle endurance and mitochondrial function after chronic normobaric hypoxia: contrast of respiratory and limb muscles. Pflugers Arch. 463:327–338. 22113781 10.1007/s00424-011-1057-8 PMC3274731

[phy212780-bib-0020] Gilliam, L. A. A. , K. H. Fisher‐Wellman , C. T. Lin , J. M. Maples , B. L. Cathey , and P. D. Neufer . 2013 The anticancer agent doxorubicin disrupts mitochondrial energy metabolism and redox balance in skeletal muscle. Free Radic. Biol. Med. 65:988–996. 24017970 10.1016/j.freeradbiomed.2013.08.191 PMC3859698

[phy212780-bib-0021] Go, A. S. , G. M. Chertow , D. Fan , C. E. McCulloch , and C. Y. Hsu . 2004 Chronic kidney disease and the risks of death, cardiovascular events, and hospitalization. N. Engl. J. Med. 351:1296–1305. 15385656 10.1056/NEJMoa041031

[phy212780-bib-0022] Granata, S. , G. Zaza , S. Simone , G. Villani , D. Latorre , P. Pontrelli , et al. 2009 Mitochondrial dysregulation and oxidative stress in patients with chronic kidney disease. BMC Genom. 10:388. 10.1186/1471-2164-10-388 PMC2737002 19698090

[phy212780-bib-0023] Himmelfarb, J. , P. Stenvinkel , T. A. Ikizler , and R. M. Hakim . 2002 The elephant in uremia: oxidant stress as a unifying concept of cardiovascular disease in uremia. Kidney Int. 62:1524–1538. 12371953 10.1046/j.1523-1755.2002.00600.x

[phy212780-bib-0024] Ide, T. , H. Tsutsui , S. Hayashidani , D. Kang , N. Suematsu , K. Nakamura , et al. 2001 Mitochondrial DNA damage and dysfunction associated with oxidative stress in failing hearts after myocardial infarction. Circ. Res. 88:529–535. 11249877 10.1161/01.res.88.5.529

[phy212780-bib-0025] Isoyama, N. , A. R. Qureshi , C. M. Avesani , B. Lindholm , P. Bàràny , O. Heimbürger , et al. 2014 Comparative associations of muscle mass and muscle strength with mortality in dialysis patients. Clin. J. Am. Soc. Nephrol. 9:1720–1728. 25074839 10.2215/CJN.10261013 PMC4186520

[phy212780-bib-0026] Kadiiska, M. B. , B. C. Gladen , D. D. Baird , D. Germolec , L. B. Graham , C. E. Parker , et al. 2005 Biomarkers of Oxidative Stress Study II: are oxidation products of lipids, proteins, and DNA markers of CCl4 poisoning? Free Radic. Biol. Med. 38:698–710. 15721980 10.1016/j.freeradbiomed.2004.09.017

[phy212780-bib-0027] Kemp, G. J. , A. V. Crowe , H. K. I. Anijeet , Q. Y. Gong , W. E. Bimson , S. P. Frostick , et al. 2004 Abnormal mitochondrial function and muscle wasting, but normal contractile efficiency, in haemodialysed patients studied non‐invasively in vivo. Nephrol. Dial. Transplant. 19:1520–1527. 15004250 10.1093/ndt/gfh189

[phy212780-bib-0028] Lewis, M. I. , M. Fournier , H. Wang , T. W. Storer , R. Casaburi , A. H. Cohen , et al. 2012 Metabolic and morphometric profile of muscle fibers in chronic hemodialysis patients. J. Appl. Physiol. 112:72–78. 22016372 10.1152/japplphysiol.00556.2011 PMC3290422

[phy212780-bib-0029] López‐Armada, M. J. , R. R. Riveiro‐Naveira , C. Vaamonde‐García , and M. N. Valcárcel‐Ares . 2013 Mitochondrial dysfunction and the inflammatory response. Mitochondrion 13:106–118. 23333405 10.1016/j.mito.2013.01.003

[phy212780-bib-0030] Madamanchi, N. R. , and M. S. Runge . 2007 Mitochondrial dysfunction in Atherosclerosis. Circ. Res. 100:460–473. 17332437 10.1161/01.RES.0000258450.44413.96

[phy212780-bib-0031] Malik, A. N. , and A. Czajka . 2013 Is mitochondrial DNA content a potential biomarker of mitochondrial dysfunction? Mitochondrion 13:481–492. 23085537 10.1016/j.mito.2012.10.011

[phy212780-bib-0032] Matsushita, K. , E. K. Williams , M. L. Mongraw‐Chaffin , J. Coresh , M. I. Schmidt , F. L. Brancati , et al. 2013 The association of plasma lactate with incident cardiovascular outcomes: the ARIC study. Am. J. Epidemiol. 178:401–409. 23817916 10.1093/aje/kwt002 PMC3727342

[phy212780-bib-0033] Milne, G. L. , S. C. Sanchez , E. S. Musiek , and J. D. Morrow . 2007 Quantification of F2‐isoprostanes as a biomarker of oxidative stress. Nat. Protoc. 2:221–226. 17401357 10.1038/nprot.2006.375

[phy212780-bib-0034] Morley, J. E. , B. Vellas , van Kan G. A. , S. D. Anker , J. M. Bauer , R. Bernabei , et al. 2013 Frailty consensus: a call to action. J. Am. Med. Dir. Assoc. 14:392–397. 23764209 10.1016/j.jamda.2013.03.022 PMC4084863

[phy212780-bib-0035] Morrow, J. D. , K. E. Hill , R. F. Burk , T. M. Nammour , K. F. Badr , and L. J. Roberts . 1990 A series of prostaglandin F2‐like compounds are produced in vivo in humans by a non‐cyclooxygenase, free radical‐catalyzed mechanism. Proc. Natl Acad. Sci. U. S. A. 87:9383–9387. 2123555 10.1073/pnas.87.23.9383 PMC55169

[phy212780-bib-0036] O'Leary, M. F. , A. Vainshtein , S. Iqbal , O. Ostojic , and D. A. Hood . 2013 Adaptive plasticity of autophagic proteins to denervation in aging skeletal muscle. Am. J. Physiol. Cell Physiol. 304:C422–C430. 23220115 10.1152/ajpcell.00240.2012

[phy212780-bib-0037] Pastoris, O. , R. Aquilani , P. Foppa , G. Bovio , S. Segagni , P. Baiardi , et al. 1997 Altered muscle energy metabolism in post‐absorptive patients with chronic renal failure. Scand. J. Urol. Nephrol. 31:281–287. 9249894 10.3109/00365599709070349

[phy212780-bib-0038] Rao, M. , L. Li , C. Demello , D. Guo , B. L. Jaber , B. J. G. Pereira , et al. and the HEMO Study Group . 2009 Mitochondrial DNA injury and mortality in hemodialysis patients. J. Am. Soc. Nephrol. 20:189–196. 18684894 10.1681/ASN.2007091031 PMC2615717

[phy212780-bib-0039] Sarnak, M. J. , and A. S. Levey . 1999 Epidemiology of cardiac disease in dialysis patients. Semin. Dial. 12:69–76.

[phy212780-bib-0040] Shah, A. J. , V. Sahgal , A. P. Quintanilla , V. Subramani , H. Singh , and R. Hughes . 1983 Muscle in chronic uremia–a histochemical and morphometric study of human quadriceps muscle biopsies. Clin. Neuropathol. 2:83–89. 6851301

[phy212780-bib-0041] Tamaki, M. , K. Miyashita , S. Wakino , M. Mitsuishi , K. Hayashi , and H. Itoh . 2014 Chronic kidney disease reduces muscle mitochondria and exercise endurance and its exacerbation by dietary protein through inactivation of pyruvate dehydrogenase. Kidney Int. 85:1330–1339. 24284514 10.1038/ki.2013.473

[phy212780-bib-0042] Terman, A. , B. Gustafsson , and U. T. Brunk . 2006 The lysosomal‐mitochondrial axis theory of postmitotic aging and cell death. Chem. Biol. Interact. 163:29–37. 16737690 10.1016/j.cbi.2006.04.013

[phy212780-bib-0043] Terman, A. , T. Kurz , M. Navratil , E. A. Arriaga , and U. T. Brunk . 2009 Mitochondrial turnover and aging of long‐lived postmitotic cells: the mitochondrial‐lysosomal axis theory of aging. Antioxid. Redox Signal. 12:503–535. 19650712 10.1089/ars.2009.2598 PMC2861545

[phy212780-bib-0044] Thompson, C. H. , G. J. Kemp , D. J. Taylor , J. G. G. Ledingham , G. K. Radda , and B. Rajagopalan . 1993 Effect of chronic uraemia on skeletal muscle metabolism in man. Nephrol. Dial. Transplant. 8:218–222. 8385287

[phy212780-bib-0045] Yen, H. C. , T. D. Oberley , C. G. Gairola , L. I. Szweda , and D. K. St. Clair . 1999 Manganese superoxide dismutase protects mitochondrial complex I against adriamycin‐induced cardiomyopathy in transgenic mice. Arch. Biochem. Biophys. 362:59–66. 9917329 10.1006/abbi.1998.1011

[phy212780-bib-0046] Yeung, C. K. , F. T. Billings , A. J. Claessens , B. Roshanravan , L. Linke , M. B. Sundell , et al. 2015 Coenzyme Q10 dose‐escalation study in hemodialysis patients: safety, tolerability, and effect on oxidative stress. BMC Nephrol. 16:1–8. 10.1186/s12882-015-0178-2 PMC4630830 26531095

[phy212780-bib-0047] Zhang, H. , M. Bosch‐Marce , L. A. Shimoda , Y. S. Tan , J. H. Baek , J. B. Wesley , et al. 2008 Mitochondrial autophagy is an HIF‐1‐dependent adaptive metabolic response to hypoxia. J. Biol. Chem. 283:10892–10903. 18281291 10.1074/jbc.M800102200 PMC2447655

[phy212780-bib-0048] Zick, M. , R. Rabl , and A. S. Reichert . 2009 Cristae formation‐linking ultrastructure and function of mitochondria. Biochim. Biophys. Acta 1793:5–19. 18620004 10.1016/j.bbamcr.2008.06.013

